# Differences in the Microbial Composition and Function of the *Arundo donax* Rhizosphere Under Different Cultivation Conditions

**DOI:** 10.3390/microorganisms12122642

**Published:** 2024-12-19

**Authors:** Fan Yang, Miaomiao Liu, Xin Wang, Yuting Hong, Qiuju Yao, Xiaoke Chang, Gongyao Shi, Weiwei Chen, Baoming Tian, Abeer Hegazy

**Affiliations:** 1 Institute of Vegetables, Henan Academy of Agricultural Sciences, Graduate T&R Base of Zhengzhou University, Zhengzhou 450002, China; xiaoyuefuxiang@163.com (F.Y.); liumm2023@163.com (M.L.); wx269152874@163.com (X.W.); hongyt182808@163.com (Y.H.); wgqdaisy@163.com (Q.Y.); cxk8802@163.com (X.C.); 2 Agricultural College, Zhengzhou University, Zhengzhou 450001, China; shigy@zzu.edu.cn (G.S.); weiwei_chen15134@zzu.edu.cn (W.C.); 3 National Water Research Center, Shubra El Kheima 13766, Egypt

**Keywords:** *Arundo donax*, high-throughput sequencing, rhizosphere microbial community

## Abstract

Rhizosphere microorganisms play an important role in the health and development of root systems. Investigating the microbial composition of the rhizosphere is central to understanding the inter-root microbial function of *Arundo donax* under various cultivation conditions. To complement the metagenomic study of the *Arundo donax* rhizosphere, here, an amplicon-based metagenomic survey of bacteria and fungi was selected as a practical approach to analyzing the abundance, diversity index, and community structure of rhizosphere bacteria and fungi, as well as to study the effects of different cultivation methods on rhizosphere microbial diversity. Next-generation sequencing and QIIME2 analysis were used. The results indicated that microbial community richness, diversity, and evenness of the hydroponic samples were lower than those of soil samples when examining the α diversity indices of bacteria and fungi using Chao1, ACE, and Shannon metrics. In particular, the relative abundances of *Proteobacteria*, *Rhizobiales*, and *Incertae sedis* in hydroponic materials were higher, while *Basidiomycota*, *Ascomycota*, and *Actinobacteriota* dominated the flora in soil materials when comparing the numbers of OTUs and the ACE community richness estimator. Furthermore, the rhizosphere of hydroponic *A. donax* contained a higher abundance of nitrogen-fixing bacteria and photosynthetic bacteria, which contribute to root formation. Additionally, there was a significant presence of *Basidiomycota*, *Ascomycota*, and *Actinobacteriota* in soil *A. donax*, which can form hyphae. This reveals that the microbial community composition of the *A. donax* rhizosphere is significantly different under various cultivation conditions, suggesting that employing two distinct culturing techniques for *Arundo donax* may alter the microbiome. Furthermore, it provides technical support for the synergistic interaction between *Arundo donax* and rhizosphere microorganisms so as to better use the relationship between *Arundo donax* and basic microorganisms to solve the problems of *Arundo donax* growth and ecological restoration.

## 1. Introduction

*Arundo donax* (*Arundo donax* L.) [1] is a perennial rhizomatous plant belonging to the Poaceae family, and it has a wide range of applications [2]. Firstly, *A. donax* is a potential energy crop. It can be burned directly as fuel, yielding substantial amounts. Research indicates that the combustion calorific values of *A. donax*, along with other energy plants such as arundinacea, switchgrass, and miscanthus, are comparable [2,3,4]. Furthermore, the carbon monoxide content in the flue gas produced by the burning of *A. donax* is lower than that of corncob, while its nitrogen oxide emissions are only slightly higher than those from pine grain, affirming its potential as an energy source [5]. Secondly, the leaves of *A. donax* contain notable levels of crude protein (20.92%), total N (3.35%), and crude fat (3.30%), making them suitable for livestock feed [6]. Studies have also demonstrated that *A. donax* has a high fiber content and, as such, can be utilized as winter roughage for animals [7]. Thirdly, *A. donax* exhibits considerable medicinal value. Research has shown that N-acetyl-D-glucosamine lectins found in the rhizomes of *A. donax* can inhibit the proliferation of human tumor cells [8] and possess pharmacological activities related to the cardiovascular system; for instance, extracts from rhizomes have been noted for their antihypertensive and anticonvulsant effects [9]. Lastly, *A. donax* plays a role in ecological restoration. The discharge of untreated domestic sewage, agricultural sewage, and industrial wastewater can lead to significant water pollution. Experiments involving constructed subsurface flow systems utilizing *A. donax* var. *versicolor* have demonstrated the effective removal of organic matter (OM) and N from waste water [10]. This plant can be employed not only for the remediation of polluted water bodies but also for the restoration of heavy-metal-contaminated soils. On the one hand, *A. donax* can absorb, fix, and transform heavy metals, such as As, Cd, and Pb, in soil through its root system, reducing the effective contents of these heavy metals in soil. On the other hand, the root system of *A. donax* has strong interception and induction effects on heavy metals, which can reduce the leakage of heavy metals into deep soil. Evidence suggests that *A. donax* can enrich heavy metals such as cadmium, lead, chromium, manganese, copper, and nickel, making it an ideal plant for the remediation of contaminated soils [11,12,13,14]. Due to its inability to produce normal functional male and female gametes, *A. donax* reproduces asexually; its seeds are sterile, and the conventional breeding method relies on tillering, resulting in an average reproductive coefficient of only 2.9–3.1 [15,16].

There exists a significant interaction between plants and microorganisms, which can yield both positive and negative effects, including symbiosis, parasitism, and antagonism [17]. Positive plant–microbe interactions such as those involving Rhizobiales, plant-growth-promoting rhizosphere bacteria (PGPR), and mycorrhiza lead to beneficial outcomes such as enhanced growth promotion, improved nutrient accessibility, and increased protection against abiotic and biological environmental stresses [18,19,20]. The root system serves as a crucial interface between the plant and soil, with the root system architecture (RSA) and growth status determining the efficiency of nutrient uptake and the capacity of plants to respond to environmental stresses [21]. In recent years, the role of rhizosphere microorganisms in regulating plant root architecture has garnered considerable attention [22,23,24]. The rhizosphere environment is a primary zone for the exchange of information and substances between plants, microorganisms, and soil [25], and rhizosphere microbes significantly influence biochemical cycling processes within the soil, playing a critical role in plant health and development [26]. These microorganisms can induce and modify root architectural traits, such as lateral roots and primary roots, through the production of hormones such as auxin (IAA), cytokinin (CTK), and strigolactones (SLs), which, in turn, positively affect plant growth [27]. Additionally, microorganisms can synthesize a variety of volatile organic compounds (VOCs) that promote root growth [28]. Rhizosphere microbes also play a crucial role in phosphate solubilization and nitrogen fixation, and they indirectly influence plant root configuration by regulating mineral nutrients [29,30]. These microbes affect the plant root structure by impacting soil aggregates and pore structure, thus indirectly influencing root configuration [31]. Finally, rhizosphere microbes can regulate soil pH, which further enhances the plant root configuration [32]. Acting as the first line of defense for plants, rhizosphere microbes enable plants to protect themselves from infection by recruiting plant-growth-promoting and pathogen-antagonistic microbes through root secretions or the immune system, thereby remodeling the rhizosphere microbiome [33,34]. This interaction is considered one of the most representative and significant influences on plant-associated microorganisms [35]. The soil microbiome is the key link in the aboveground–underground interactions of terrestrial ecosystems and plays a key role in plant health by taking part in the processes of plant nutrient acquisition and soil nutrient cycling [36].

The composition of the *A. donax* rhizosphere changes under different physiological conditions, which helps the plant cope with changes in the environment [37,38]. Exogenous microbial agents can help *A. donax* cope with and improve its growing environment [39,40]. It has also been found that the rhizosphere microorganisms of *A. donax* exhibit no obvious changes under the influence of sludge [41]. However, whether the composition of the *A. donax* rhizosphere changes when the plant is grown in a hydroponic system as opposed to in soil remains unknown. The aim of this study was to investigate the rhizosphere microbial composition, community structure, and dominant microbial population function of *A. donax* under different cultivation conditions. One is hydroponic, under which conditions the *A. donax* can prevent wind erosion, stabilize sandy soil, and remediate sewage. The other is soil, under which conditions the *A. donax* can be used as an energy crop, fodder for livestock, and for the remediation of polluted soil. In this study, the rhizosphere microbiome was analyzed under two different models, which revealed the dominant components of the microbial population and provided technical support for the suggested functional interactions between *A. donax* and rhizosphere microbes. This research is important in order to make better use of the relationship between *A. donax* and rhizosphere microorganisms to solve the problems associated with *A. donax* growth and ecological restoration.

## 2. Materials and Methods

### 2.1. Sample Collection

The materials used in this experiment included *A. donax* cultivated at the College of Agriculture, Zhengzhou University. All the materials were grown in the natural environment. Soil samples were collected using the five-point random sampling method. A soil drill was employed to collect rhizosphere soil and root tissue from 20 cm below the soil surface, which were then placed in aseptic bags and labeled accordingly. The hydroponic materials selected were uniform in size, exhibited healthy growth, and were maintained under identical growth conditions. Here, 1–2 cm of root and surface water were placed in a sterile bag and labeled. After all samples were collected, they were stored in ice packs and transported to the laboratory within 12 h. A portion of the rhizosphere soil samples was stored at −80 °C until DNA extraction, and a portion was immediately used for physicochemical analysis.

### 2.2. 16S-rRNA Gene and Ribosomal DNA ITS Region-End Amplification and Sequencing

Bacterial DNA samples were extracted using the CretMagTM Plant DNA Mini Kit. The 16S-rRNA gene targeting the V3–V4 region was amplified via PCR with primers 341F and 806R under the following conditions: 95 °C for 3 min, followed by 30 cycles of 95 °C for 30 s, 55 °C for 30 s, 72 °C for 45 s, and 72 °C for 10 min. Fungal DNA samples were extracted using the CretMagTM Power Soil DNA Kit (CretBiotech, Shanghai, China). The hypervariable region of the ITS was amplified with the primer pairs ITS1F and ITS2R using an A200 PCR thermocycler. PCR amplification was performed as follows: initial denaturation at 94 °C for 2 min, followed by 30 cycles of denaturing at 94 °C for 30 s; annealing at 55 °C for 30 s and extension at 72 °C for 45 s; a single extension at 72 °C for 10 min; and ending at 4 °C. Thereafter, the target band size was verified through 1% agarose gel electrophoresis and purified using the Agencourt AMPure XP Nucleic Acid Purification Kit (Axygen Biosciences, Union City, CA, USA). Sequencing was performed on the Illumina NovaSeq platform (Illumina, San Diego, CA, USA), generating 250 bp paired-end reads.

### 2.3. Bioinformatic Analysis

The present study employed Trimmomatic(v0.36) and Pear(v0.9.6) for the quality control of the Fastq data. In Trimmomatic, a sliding window strategy was adopted, with a window size of 50 bp, an average quality score of 20, and a minimum sequence length of 120 bp. Pear was utilized to eliminate sequences containing “N”. Flash and Pear were employed to merge the paired-end sequences based on their overlap, with a minimum overlap set to 10 bp and a maximum mismatch ratio of 0.1 to obtain Fasta sequences. The chimeras in the Fasta dataset were compared against a known database using the uchime method. For the unknown database, the de novo method was applied to remove short sequences, ultimately yielding high-quality sequences. The OTU clustering of non-repetitive sequences was performed, during which chimeric sequences were removed, resulting in the representative OTU sequences. Following this, analyses were conducted using the QIIME2 sequencing platform (Illumina, San Diego, CA, USA), including alpha diversity analysis, species annotation, beta diversity analysis, correlation analysis, and advanced analysis of the data. The advanced analysis of data included PICRUSt analysis and FAPROTAX analysis. All the raw sequence data have been made available in the NCBI (Bethesda, MD, USA) Sequence Read Archive (SRA) database under the bioproject accession number PRJNA1135131.

## 3. Results

### 3.1. Effects of Different Cultivation Conditions on the Diversity and Community Composition of Rhizosphere Bacteria of Arundo donax

#### 3.1.1. High-Throughput Sequencing Data and Diversity Analysis of Rhizosphere Bacteria

**Figure 1:**
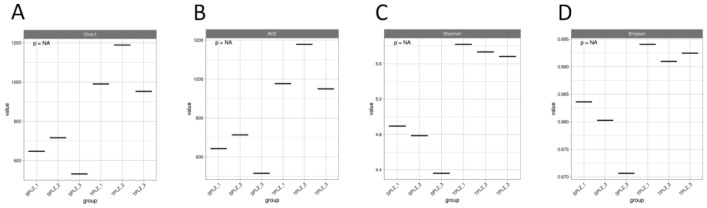
Alpha diversity index box plots. Box plots show the variations in Chao1 (**A**), ACE (**B**), Shannon (**C**), and Simpson (**D**) indices for bacteria (*p* < 0.05).

A high-throughput sequencing analysis of rhizosphere microorganisms was conducted using the QIIME2 sequencing platform. After flattening the minimum sample, a total of 4948 OTUs were identified through OTU division and species annotation. A dilution curve was generated by randomly sampling a specific number of sequences from each sample. The result indicated that the curve gradually tended to flatten, suggesting that the quantity of sequencing data was reasonable and adequate to reflect the majority of microbial diversity information present in the samples. Those with the lowest sequence numbers were analyzed to determine their alpha diversity index (Figure 1). Soil culture metrics, including the Chao1, ACE, and Shannon indices, revealed an increase in bacterial alpha diversity, indicating that the microbial community richness, diversity, and evenness in the hydroponic samples were lower than those in the soil samples.

#### 3.1.2. Community Composition and Dominant Species Abundance of Rhizosphere Bacteria

**Figure 2:**
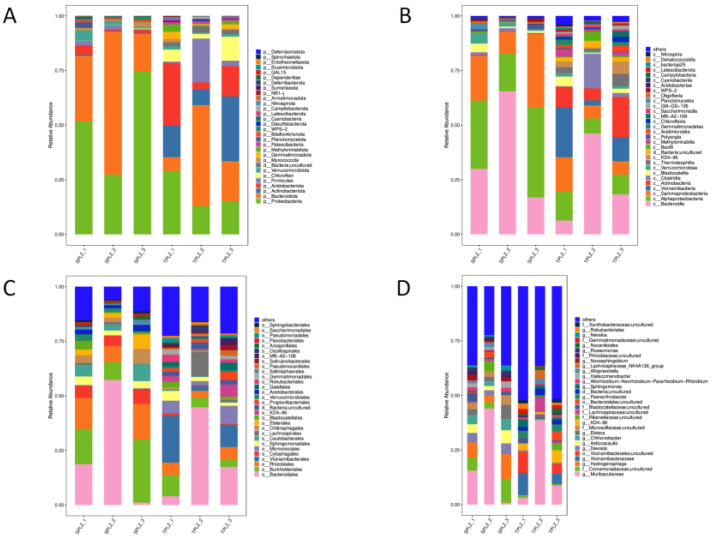
Bacterial community composition and relative abundance in the rhizosphere of *Arundo donax* under different cultivation methods at the phylum (**A**), class (**B**), order (**C**), and genus (**D**) levels (top 30). The horizontal coordinate is the sample name, and the vertical coordinate is the relative abundance of the species in the sample. The figure shows information for species with a relative abundance of more than 1%.

The bacterial community composition in the rhizosphere of *A. donax* grown under different culture conditions is shown in Figure 2. There were 30 bacterial phyla with relative abundance exceeding 1%, and the six phyla with the highest percentage of bacteria were, in order, *Proteobacteria*, *Bacteroidota*, *Actinobacteriota*, *Acidobacteriota*, *Firmicutes*, and *Chloroflexi*. The relative abundance of *Proteobacteria* and *Bacteroidota* in the hydroponic materials was higher than that in the soil materials, while the relative abundance of *Actinobacteriota* was lower than that in the soil materials (Figure 2A). At the class level, *Bacteroides*, *Alphaproteobacteria*, and *Gammaproteobacteria* accounted for the largest proportion of bacteria in the hydroponic materials. In addition to the above three classes, *Vicinamibacteria* and *Actinobacteriota* accounted for the largest proportion of bacteria in the soil culture materials. The relative abundance of *Alphaproteobacteria* and *Gammaproteobacteria* in the hydroponic materials was higher than that in the soil, while the relative abundance of *Vicinamibacteria* was significantly lower (*p* < 0.05) than that in the soil (Figure 2B). The dominant bacteria in the rhizosphere of *Arundo donax* were mainly *Bacteroidales*, *Burkholderiales*, and *Rhizobiales*. The relative abundance of *Rhizobiales* in the hydroponic materials was higher than that in the soil, while the relative abundance of *Cytophagales* was lower than that in the soil (Figure 2C). At the genus level, the relative abundance of *Hydrogenophaga* in the hydroponic materials was significantly higher (*p* < 0.05) than that in the soil materials (Figure 2D).

#### 3.1.3. Clustering Analysis of Bacterial Community and Similarity and Difference in Its Structure

**Figure 3:**
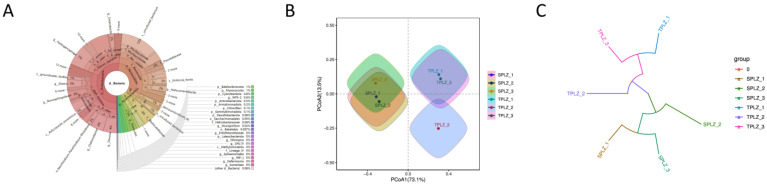
Krona sample illustration (**A**). The circles represent the different classification levels from the inside to the outside, and the fan size represents the relative proportion of different OTU annotation results. Analysis of differences between samples. PCoA analysis diagram (**B**). The first principal component and its contribution to the difference in samples are shown in the horizontal coordinates, and the second principal component and its contribution to the difference in samples are shown in the vertical coordinates. Based on the Bray–Curtis multi-sample clustering tree (**C**). The length of the branches represents the distance between the samples, and the more similar the samples, the more likely they are to be clustered together.

Krona tools were used to visualize the results of species annotations (Figure 3A). In the displayed results, the circles represent the different classification levels from the inside to the outside, and the fan size represents the relative proportion of different OTU annotation results. The results showed that the relative abundances of *Proteobacteria* and *Rhizobiales* in the hydroponic materials were higher than in the soil, while the relative abundance of *Actinobacteriota* was lower than in the soil.

Principal coordinate analysis (PCoA) was used to assess the similarities and differences in the structure of the rhizosphere bacterial community of *A. donax* under different culture conditions. The results showed that the hydroponic group and the soil group could be clearly distinguished on the first axis, and the corresponding microbial community composition and structure were significantly different (Figure 3B). Hierarchical clustering analysis was performed based on the beta diversity distance matrix, and the tree structure was constructed using the UPGMA algorithm to obtain a heatmap. Based on the Bray–Curtis multi-sample cluster tree, it was also shown that the rhizosphere bacterial community structure differed under different culture conditions (Figure 3C).

#### 3.1.4. Prediction of Metabolic Functions of the Rhizosphere Bacteria

**Figure 4:**
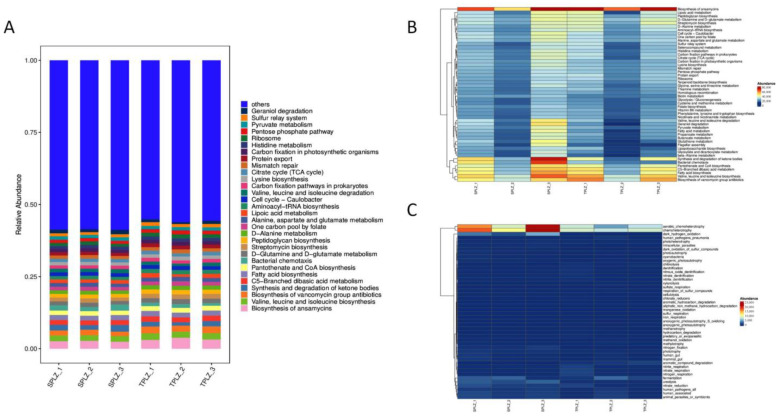
Functional metabolism prediction. Column chart of functional abundance (**A**): horizontal coordinate is the sample name, and vertical coordinate is the proportion. Functional abundance cluster heatmap (**B**,**C**). Horizontal coordinates for different samples; vertical coordinates for the first 50 abundances of the function.

Based on the 16S rDNA information for bacteria and the OTU information for relative species after a comparison with the GreenGene database, functional abundance maps of *A. donax* material were obtained by predicting the metabolic functions of bacterial colonies using the PICRUSt tool (Figure 4A). The results of the PLCRUST analysis showed that there was no significant difference in bacterial metabolic function between the hydroponic and soil-cultured materials. The biosynthesis of ansamycins and valine, leucine and isoleucine biosynthesis, the biosynthesis of vancomycin group antibiotics, the synthesis and degradation of ketone bodies, and C5-branched dibasic acid metabolism are relatively high. The heatmap results show that the synthesis and degradation of ketone bodies and bacterial chemotaxis in the hydroponic materials were higher than those in the soil (Figure 4B). The relative abundances of aerobic chemoheterotrophy, chemoheterotrophy, and dark hydrogen oxidation were also higher in the hydroponic materials than those in the soil culture (Figure 4C).

### 3.2. Effects of Different Cultivation Conditions on the Diversity and Community Composition of Rhizosphere Fungi of A. donax

#### 3.2.1. High-Throughput Sequencing Data and Diversity Analysis of Rhizosphere Fungi

**Figure 5:**
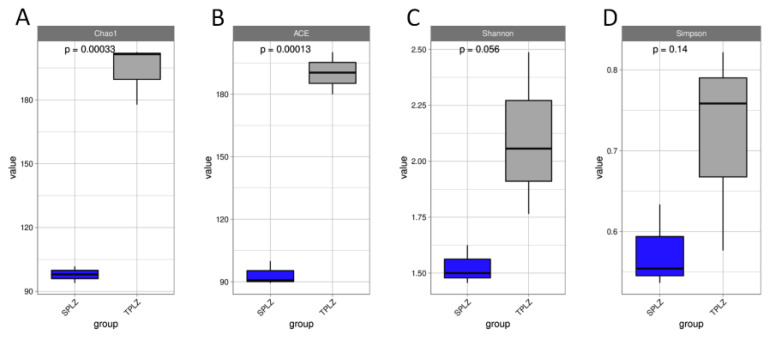
Alpha diversity index box plot. Box plots show the variation in Chao1 (**A**), ACE (**B**), Shannon (**C**), and Simpson (**D**) indices for fungi (*p* < 0.05).

A high-throughput sequencing analysis of the rhizosphere microorganisms was carried out using the QIIME2 sequencing platform. In total, 756,116 effective sequences were obtained after optimized quality control. A total of 840 OTUs were obtained through OTU division and species annotation after the minimum sample sequence number was flattened. The gradual flattening of the *Specaccum* species accumulation curve suggests a reasonable amount of sequencing data to reflect the vast majority of microbial diversity information in the sample. Rank-abundance curves were drawn by ranking the OTU abundance levels. The results showed that the curve for the soil culture materials was large and smooth on the horizontal axis, indicating that the rhizosphere fungal communities of the soil culture materials were abundant and distributed evenly. The number of samples with the lowest sequence number was analyzed to obtain its fungi diversity index (Figure 5). Chao1 index, ACE index, and Shannon index showed an increase in bacterial alpha diversity, indicating that the microbial community richness, diversity, and evenness of the hydroponic samples were lower than those of the soil culture.

#### 3.2.2. Community Composition and Dominant Species Abundance of Rhizosphere Fungi

**Figure 6:**
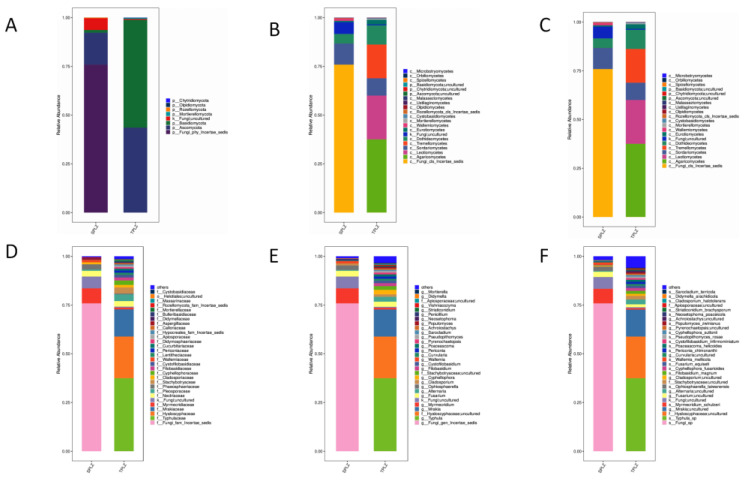
The composition and relative abundance of fungal communities in the rhizosphere of *Arundo donax* were assessed under various cultivation methods at the phylum (**A**), class (**B**), order (**C**), family (**D**), genus (**E**), and species (**F**) levels. The horizontal coordinate is the sample name, and the vertical coordinate is the relative abundance of the species in the sample. The figure shows species information with a relative abundance of more than 1%.

The species composition of rhizosphere fungi associated with *A. donax*, examined using different culture methods at the phylum level, revealed the presence of six fungi phyla from six rhizosphere samples; among these, *Incertae sedis*, *Basidiomycota*, and *Ascomycota* were the dominant fungi in the rhizosphere (Figure 6A). There were obvious differences in the distribution of the fungal community across the various culture methods. The relative abundance of *Incertae sedis* in the hydroponic materials was significantly higher than in the soil, whereas the relative abundance of *Basidiomycota* was lower under hydroponic conditions compared with the soil culture. At the class level, 21 fungal classes were annotated. The hydroponic materials were predominantly characterized by *Incertae sedis*, which exhibited a significantly higher relative abundance than in the soil materials, while the relative abundance of *Agaricomycetes* was significantly lower in the hydroponic materials (Figure 6B). At the order level, the relative abundance of *Incertae sedis* in the hydroponic materials was also significantly higher than that in the soil, while the relative abundances of *Agaricales* and *Helotiales* in the hydroponic materials were significantly lower in the hydroponic materials than in the soil (Figure 6C). At the family level, *Incertae sedis* showed a significantly higher relative abundance in the hydroponic materials compared with the soil, while the relative abundances of *Typhulaceae* and *Hyaloscyphaceae* were significantly lower under hydroponic conditions (Figure 6D). At the genus level, *Incertae sedis* emerged as the most important genus of the fungi identified in the hydroponic materials. The largest proportion of soil-cultured fungi was found in the genus *Typhula* (Figure 6E).

#### 3.2.3. Clustering Analysis of the Fungal Communities and Their Structural Similarities and Differences

**Figure 7:**
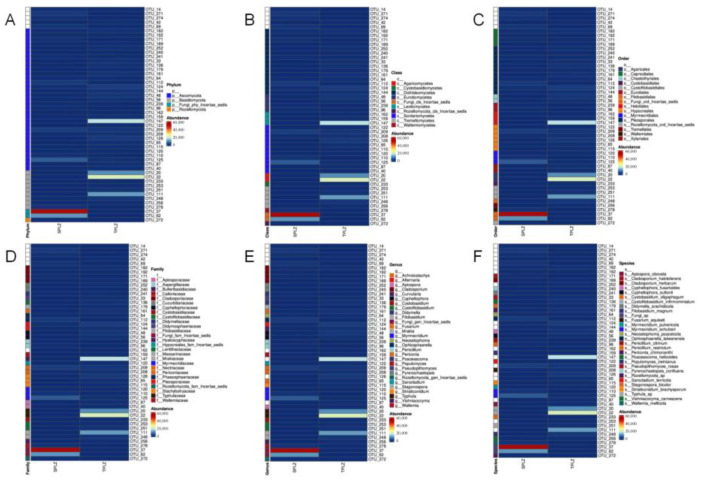
Heatmap of OTUs and their corresponding taxonomic levels presented for the phylum (**A**), class (**B**), order (**C**), family (**D**), genus (**E**), and species (**F**).

Heatmaps were generated based on species annotation and abundance data for the rhizosphere microorganisms (Figure 7), and clustering was performed using two levels of taxonomic information and inter-sample differences. The results indicated that the rhizosphere fungal communities exhibited variation among the different cultivation methods. Notably, *Incertae sedis* was dominant in the hydroponic culture, whereas *Basidiomycota* predominated in the soil culture.

**Figure 8:**
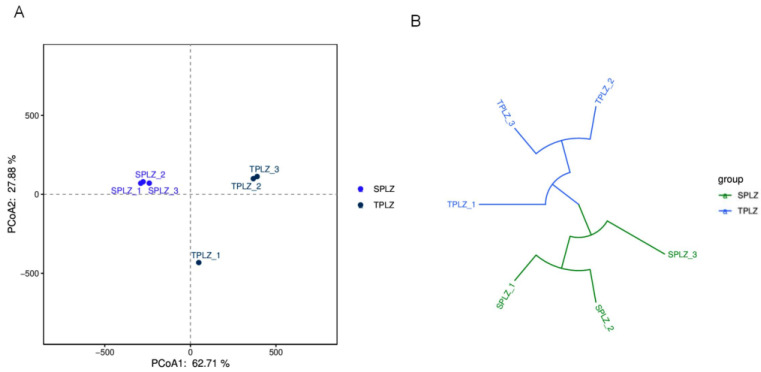
Analysis of differences between samples illustrated through a PCoA analysis diagram (**A**) and a Bray–Curtis multi-sample clustering tree (**B**). The points indicate the species composition of each sample: the horizontal coordinates indicate the first principal component and its contribution to the difference in samples; the vertical coordinates indicate the second principal component and its contribution to the difference in samples. The length of the branches represents the distance between the samples, and the more similar the samples, the more likely they are to be clustered together.

Principal coordinate analysis (PCoA) was conducted at the OTU level to examine the rhizosphere fungal communities across two taxonomic groups and six samples. Principal coordinate analysis maps were generated to evaluate the similarities and differences in the rhizosphere fungal communities under varying culture conditions, as illustrated in Figure 8A. The first and second principal coordinates, PCoA1 and PCoA2, accounted for 62.71% and 27.88% of the variance in the fungal community, respectively. The three hydroponic samples clustered closely within the same quadrant, indicating the fungal community structure. In contrast, the hydroponic and soil samples were positioned far apart in the figure, suggesting a significant difference in the fungal community structure between these two conditions. Additionally, the Bray–Curtis multi-sample clustering tree further confirmed the distinct rhizosphere fungal community structure associated with different culture methods, as shown in Figure 8B.

#### 3.2.4. Prediction of the Metabolic Functions of Rhizosphere Fungal Species

**Figure 9:**
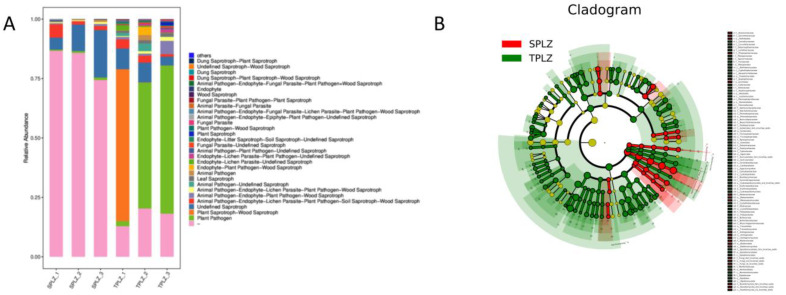
Column chart illustrating functional abundance (**A**) and cladogram presenting the distribution and cladistics of LDA values for various species (**B**). The circles radiating from the inside to the outside of the branching graph represent the taxonomic level from phylum to genus (or species). Each small circle at a different classification level represents a classification at that level, and the diameter of the small circle is proportional to the relative abundance. The non-significantly different species are uniformly colored in yellow, with red nodes representing the microbiota playing an important role in the red group and green nodes representing the microbiota playing an important role in the green group. The other circle colors have the same meaning.

A FUNGuild analysis was conducted and revealed significant differences (*p* < 0.05) in fungal metabolic functions between the hydroponic and soil-cultured materials. Specifically, the plant–pathogen and plant saprotroph–wood saprotroph categories in the soil-cultured materials were significantly higher (*p* < 0.05) than those in the hydroponic materials, while the latter exhibited significantly lower values (Figure 9A). Furthermore, a LEfSe analysis corroborated these findings, indicating substantial differences in the metabolic functions of fungi between the hydroponic and soil-cultured materials (Figure 9B).

## 4. Discussion

To investigate the effects of cultivation conditions on *A. donax*, we studied the rhizosphere microbial composition. Principal coordinate analysis based on Bray–Curtis multi-sample clustering demonstrated that the rhizosphere bacterial community structure of *A. donax* cultivated in soil exhibited greater richness compared with those grown hydroponically, as indicated by increased Chao1, ACE, and Shannon diversity indices. Furthermore, the relative abundance of *Proteobacteria* and *Rhizobiales* in hydroponic materials was higher than that in soil culture materials, while *Acidobacteria* and *Actinomycetes* in the soil culture materials were the dominant bacterial groups. Consequently, this study provides essential insights into the structure and function of rhizosphere microbes. 

In China, the constraints imposed by the Red Line limit cropland to an area of 1.2 × 10^12^ m^2^. The Red Line is a strict land use law designed to protect a minimum amount of arable land to ensure food security, reflecting China’s concerns about balancing urbanization, industrialization, and environmental sustainability with the need to feed its population. This, coupled with the imbalance between supply and demand for forage grain, highlight the potential of high-yield *A. donax* as a source of carbon sink plants. These plants can contribute to energy savings and emission reductions while also serving as excellent forage plant options [42], positioning this crop as a promising new energy resource with significant development potential [43]. In addition, *A. donax* can also be planted in contaminated soil and metal-contaminated sites and waters for phytoremediation [44,45]. Studies have shown that microbes can help *A. donax* improve bioremediation systems to remove clarithromycin (CLA) and diclofenac (DCF) [44]. Hydroponic *A. donax* has greater potential for cadmium remediation [45]. Therefore, the microbial changes in different environments may be beneficial to the development of the phytoremediation function of *A. donax*, which is consistent with the results of this experiment.

Our finding that the *A. donax* rhizosphere contains a high abundance of *Actinobacteriota* suggests that it is particularly effective in the biological treatment of sewage and organic solid waste, as well as in soil improvement. *Actinobacteriota*, which have been identified in soil-cultured *A. donax*, are widely distributed in nature as spores or hyphae, particularly in soils characterized by low water content, abundant organic matter (OM), and neutral to slightly alkaline pH [46]. This microorganism plays a crucial role in the breakdown of various organic compounds, including aromatic compounds, paraffin, rubber, cellulose, lignin, and other complex molecules, as well as some highly toxic substances such as cyanide [47]. Consequently, they are not only active in the natural material cycle but also contribute to the biological treatment of sewage and organic solid waste [48], thereby promoting soil aggregate formation and enhancing soil quality [49]. Acidobacteria can be enriched in acid soils with heavy metal pollution and may have the metabolic potential to tolerate or even transform heavy metals, which is helpful in the remediation of soil heavy metal pollution [50]. Consequently, this study provides a scientific basis for enhancing the ecological restoration ability of *Arundo donax* by utilizing its relationship with rhizosphere microorganisms.

Rhizosphere microbes serve as the first line of defense for plants, enabling them to protect themselves from infections by recruiting plant-growth-promoting and pathogen-antagonistic microbes through root secretions or the immune system remodeling of the rhizosphere microbiome [33,34], which is considered to be the most representative and most closely affected plant microorganisms [35]. Fungi represent a group of soil microorganisms that serve as primary decomposers, regulating the decomposition of organic matter in the soil and, consequently, exerting a direct influence on plant growth and development [51]. The heatmap analysis indicated significant differences (*p* < 0.05) among the rhizosphere fungal communities of *A. donax* under various cultivation methods, revealing that fungi from the *I. sedis* clade predominate in hydroponic systems. Given that *I. sedis* plays a pivotal role in productivity and the C cycle, as well as being among the most abundant photosynthetic microorganisms in the ocean [52], their colonies can alter the physical and chemical properties of the environment and degrade microplastics, thereby delaying the photoaging process [53]. In addition, research has shown that I. sedis can serve as the primary producer in hydroponic systems, producing organic matter through photosynthesis that can act as a food source for other organisms in the system [54]. Therefore, their presence in hydroponic systems is vital for enhancing photosynthesis and facilitating microplastic degradation. *Basidiomycota* and *Ascomycota* are the primary groups found in soil-cultured materials, aligning with the findings from previous studies on other plant species [55,56,57]. *Ascomycota*, primarily composed of saprophytic fungi, are recognized as the main fungi responsible for degrading complex organic matter, thus supplying essential nutrients to plants during soil nutrient cycling. *Basidiomycota* can form mycorrhizal associations with plants, which enhances plant resistance to pathogens and promotes plant growth and development [58]. Consequently, this symbiotic relationship strengthens the plant’s ability to defend itself. Therefore, this study further elucidates the differences in the ecological functions of *Arundo donax* under different cultivation conditions.

In the context of plant–pathogen and plant saprotroph–wood interactions, saprotrophs were identified as the primary functional group through FUNGuild analysis, although at a significantly lower (*p* < 0.05) level than those observed in the hydroponic materials. The ANOVA results indicated substantial differences in the metabolic functions of fungi between the hydroponic and soil-cultured materials, with the relative abundance of fungi in the hydroponic materials being significantly higher (*p* < 0.05) than that in the soil. Furthermore, the abundance of animal parasites, fungal parasites, animal pathogens, endophytes, and plant pathogens associated with wood saprotrophs in the hydroponic materials was significantly lower than that found in the soil materials.

In terms of microbial diversity, the rhizosphere of hydroponic *A. donax* exhibited a higher abundance of *Proteobacteria*, *Rhizobiales*, and *Incertae sedis*, which are associated with enhanced nitrogen fixation and photosynthetic capabilities. This species not only possesses the highest tensile strength and Young’s modulus but also demonstrates superior soil consolidation capacity, allowing it to thrive in fluctuating reservoir zones. The elevated photosynthetic intensity of *A. donax* enables it to be cultivated and harvested over extended periods, thereby satisfying industrial demands for reeds and fostering the development of new industries. Additionally, the high abundances of *Actinobacteriota*, *Basidiomycota*, and *Ascomycota* in the rhizosphere of *A. donax* significantly contribute to natural material circling, the biological treatment of sewage and organic solid waste, and the formation of soil aggregates, making it an ideal candidate for the remediation of contaminated soil.

## 5. Conclusions

The findings of the current study indicate that the microbial community structure and composition within the rhizosphere of *A. donax* is influenced by different culture conditions. Notably, in this study, the composition of the rhizosphere microbial community exhibited significant differences (*p* < 0.05) across various cultivation methods. The richness and diversity of the fungi and bacteria in the soil-cultured *A. donax* were greater than those observed in the hydroponic systems. In contrast, the abundance of Proteobacteria and Rhizobiales was higher in the hydroponic materials, with I. sedis identified as the dominant species. The high abundance of photosynthetic and N-fixing bacteria facilitated increased root production in *A. donax*. Additionally, the abundance of Actinobacteriota in the soil culture materials was elevated, with Ascomycota and Basidiomycota identified as the dominant phyla. These groups, including Basidiomycota, Ascomycota, and Actinobacteriota, contributed to the increased production of hyphae in the roots of *A. donax*.

**Figure 10:**
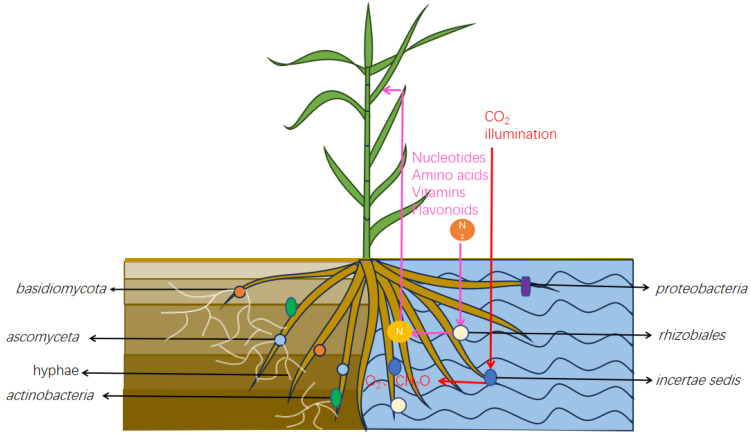
Illustration of the rhizosphere microorganism mechanism and its effect on *Arundo donax*.

In summary, this study demonstrates a synergistic interaction between *A. donax* and rhizosphere microorganisms, and the results can be used to better understand the relationship between *A. donax* and rhizosphere microorganisms to solve the problems associated with *A. donax* growth and ecological restoration. Finally, we drew a schematic diagram of the interaction between soil factors and rhizosphere microorganisms, revealing the differences in the composition and function of rhizosphere microorganisms of *A. donax* under different cultivation conditions (Figure 10).

## Data Availability

All raw sequence data have been made available in the NCBI Sequence Read Archive (SRA) database under the bioproject accession number PRJNA1135131.
